# Normative mammillary body volumes: From the neonatal period to young adult

**DOI:** 10.1016/j.ynirp.2022.100122

**Published:** 2022-12

**Authors:** Seralynne D. Vann, Cornel Zachiu, Karlijn M.E. Meys, Sara Ambrosino, Sarah Durston, Linda S. de Vries, Floris Groenendaal, Maarten H. Lequin

**Affiliations:** a School of Psychology, Neuroscience and Mental Health Research Institute, Cardiff University, Cardiff, UK; b Department of Radiotherapy, University Medical Center Utrecht, 3584 CX, Utrecht, Utrecht, the Netherlands; c Division Imaging & Oncology, Department of Radiology & Nuclear Medicine, University Medical Center Utrecht & Princess Máxima Center for Pediatric Oncology, 3508 GA, Utrecht, the Netherlands; d Department of Psychiatry, University Medical Center Utrecht Brain Center, Utrecht University, Utrecht, the Netherlands; e Education Center, Medical Center Utrecht, Heidelberglaan 100, 3584 CX, Utrecht, the Netherlands; f Deparment of Neonatology, Wilhelmina Children's Hospital, University Medical Center Utrecht, the Netherlands

## Abstract

The mammillary bodies may be small, but they have an important role in encoding complex memories. Mammillary body pathology often occurs following thiamine deficiency but there is increasing evidence that the mammillary bodies are also compromised in other neurological conditions and in younger ages groups. For example, the mammillary bodies are frequently affected in neonates with hypoxic-ischemic encephalopathy. At present, there is no normative data for the mammillary bodies in younger groups making it difficult to identify abnormalities in neurological disorders. To address this, the present study set out to develop a normative dataset for neonates and for children to young adult. A further aim was to determine whether there were laterality or sex differences in mammillary body volumes. Mammillary body volumes were obtained from MRI scans from 506 participants across two datasets. Measures for neonates were acquired from the Developing Human Connectome Project database (156 male; 100 female); volumes for individuals aged 6–24 were acquired from the NICHE database (166 males; 84 females). Volume measurements were acquired using a semi-automated multi-atlas segmentation approach. Mammillary body volumes increased up to approximately 15 years-of-age. The left mammillary body was marginally, but significantly, larger than the right in the neonates with a similar pattern in older children/young adults. In neonates, the mammillary bodies in males were slightly bigger than females but no sex differences were present in older children/young adults. Given the increasing presentation of mammillary body pathology in neonates and children, these normative data will enable better assessment of the mammillary bodies in healthy and at-risk populations.

## Highlights

• Normative volume data are presented for the mammillary bodies for neonates and for age 6–24.
• The mammillary bodies show the greatest growth trajectory during the neonatal period.
• Mammillary bodies are larger in males than females in neonates but there is no effect of sex in children/young adults.
• The left mammillary body is larger than the right mammillary body in neonates and older children/young adults.

## 1 Introduction

The mammillary bodies (MBs) may be small, but they form a key part of an extended memory network and are particularly important for encoding complex memories (McNaughton and Vann, 2022). In adults, damage to the MBs often occurs due to thiamine deficiency resulting in Korsakoff syndrome (Kopelman, 1995). In these patients, the MBs show volume loss, scarring and inflammation on histology (Mair et al., 1979). While there has been a long-standing awareness of MB pathology in adults, it is only more recently that researchers have become aware of the vulnerability of the MBs in younger populations (Meys et al., 2022); both as a result of thiamine deficiency as well as from hypoxia-ischemia. The effect of hypoxia-ischemia is particularly evident during the neonatal period where up to 40% of infants with hypoxic-ischemic encephalopathy (HIE) show an abnormal signal intensity in the MBs (Molavi et al., 2019; Lequin et al., 2021). A follow-up study in school children showed that, in the majority of cases, the HIE-related abnormal signal progressed to severe MB atrophy and this MB atrophy was also associated with poorer memory (Annink et al., 2021).

In many cases of HIE, the MB atrophy is so substantial that it is possible to make a binary decision as to whether the MBs are present or absent. But in other cases, the MBs are still visible but there may be partial atrophy. At this point, quantitative analyses are needed to determine the extent of the MB pathology. In adults, for example, MB size has been shown to be linked to recollective memory performance, with increasing impairments with decreasing MB volume; memory impairments become most apparent when the MBs are over 50% reduced in size (Tsivilis et al., 2008). It is not clear whether a similar pattern is seen in children. To help determine whether there is a cut-off point at which MB pathology can produce cognitive impairments in children, it is important to have a better understanding of the range of MB volumes in normal populations. To date, normative data for MBs has only been available for adult populations, with often a focus on older individuals. Therefore, the primary goal of this study was to provide a comprehensive reference source to help clinicians decide whether MB volumes in younger individuals are within the normal range.

A second goal of the study was to look at age-related changes in MB volume in the developing brain. Brain development, in terms of both structural growth and connectivity, is protracted and there is substantial heterogeneity in growth patterns across cortical and subcortical regions (Wierenga et al., 2014a, 2014b, 2016). While the MBs are relatively well-developed at birth (Koutcherov et al., 2003), it is not clear how they develop during the post-natal period and at what age they would be considered fully-developed in terms of size.

A final goal was to determine whether there are any lateralization effects in the MBs at a population level. Previous studies have assessed the incidence of MB asymmetry at an individual level, reporting a relatively high degree of asymmetry in healthy individuals, ranging from 6.5% to 14% (Ozturk et al., 2008; Tagliamonte et al., 2015). These studies made a qualitative assessment of whether the MBs were asymmetric, so it is unclear whether these asymmetries reflect a difference in volume, displacement, or are due to the plane in which the images are acquired. By acquiring volumetric measures of the left and right MBs separately, it is possible to determine whether there are lateralization effects at the population level but also whether there are common incidences of asymmetry (i.e., one MB abnormally smaller than the other) at an individual level.

To address these goals, bilateral MB volumes were obtained from two databases: Developing Human Connectome Project database and the NICHE database. Intracranial volumes were also acquired from the NICHE database.

## 2 Material and methods

### 2.1 Participants

#### 2.1.1 dHCP database

MB volume measurements were obtained from the scans of 256 neonatal participants (156 male, 100 female), selected from the “Developing human connectome project” (dHCP) open database (Hughes et al., 2017; Bastiani et al., 2019; Fitzgibbon et al., 2020) (http://www.developingconnectome.org/). The selection criterion was whether the imaging protocol for a particular participant included a T1-weighted MR scan (to be compatible with the data from the NICHE database – see below). The gestational age for the subjects at birth was 36.6 ± 4.4 weeks (range: 24.6–42.3 weeks); the gestational age at the time of the scan was 39.9 ± 2.4 weeks (range: 29.3–45.1 weeks). Within the cohort, 198 of the infants were born as singletons and 58 were born in multiple births. The scans were rated by a neuroradiologist as either appearing normal or showing incidental findings with unlikely significance for clinical outcome of analysis.

#### 2.1.2 NICHE database

The NICHE database includes scans from typically developing individuals in the context of various research projects conducted at the NICHE lab in the Psychiatry Department of the University Medical Center Utrecht (head: Prof. Sarah Durston). Due to the longitudinal nature of many studies at the NICHE lab, a subset of participants was scanned twice or more. For this study, only one scan per individual was included. This was typically the first scan, unless a subsequent scan was of better quality (as rated by neuroradiologists) or obtained on 3T rather than 1.5T. Scan selection was carried by SDV without knowledge of MB volume. Scans were included from 250 participants (166 males; 84 females). The age range was 6–24 (mean 13.7 ± 4.3 years).

Participants were recruited through schools and other educational centers. For participants under 18 years of age, the Diagnostic Interview Schedule for Children (DISC, version 2.3 or IV) parent version (Shaffer et al., 2000) was administered to parents by a qualified researcher to exclude psychiatric comorbidity at study entry. Older participants participated in the Mini-International Neuropsychiatric Interview to confirm the absence of psychopathology (Sheehan et al., 1998).

Participants were excluded when there was evidence of psychiatric morbidity or first-degree relatives with a history of psychiatric problems. Additional exclusion criteria were an estimated IQ below 70 on the Wechsler Intelligence Scales [WISC-R/WISC-III or WAIS-III as appropriate, Dutch versions (Wechsler, 2005)]; any major physical or neurological illnesses, or the presence of metals in the body that precluded an MRI scan. None of the participants were taking any form of psychoactive medication.

Prior to the MRI scan, children under 13 years of age were acclimatized to the MRI procedure in a practice session using a mock scanner [see (Durston et al., 2009)]; children over 13 years were also offered the opportunity to take part in a practice session.

The Institutional Review Board of the University Medical Center Utrecht, the Netherlands, approved these studies and their procedures. For participants under the age of 12 years, written informed consent was obtained from the parents after full disclosure of the study purpose and procedures. For children aged 12–16 years, parental consent was obtained in addition to individual consent. For participants aged 16 years and over, written informed consent was only obtained from the individual.

### 2.2 MRI acquisition – NICHE database

One hundred and ten T1-weighted 3-dimensional fast field echo scans of the whole head were acquired on a Philips Achieva 1.5-T scanner (Philips Medical Systems, Best, The Netherlands) with 130–150 1.5-mm contiguous coronal slices (41 scans), or 160 to 180 1.2 mm contiguous coronal slices (174 scans) (echo time 4.6 ms; repetition time 30 ms; flip angle 30°; field of view 25.6 cm; in-lane voxel size 1mm × 1 mm).

One hundred and forty whole-brain MRI 3-T scans were acquired on a 3.0-T Philips Achieva MRI-scanner, utilizing either the following T1-weighted parameters for 127 scans (echo time 4.6 ms; repetition time 10 ms; flip angle 8°, matrix 304 × 299, field of view 24 cm, voxel size 0.75 × 0.75 × 0.8 mm^3^), or the Alzheimer's Disease Neuroimaging Initiatives (ADNI) MRI protocol (Jack et al., 2008) for 13 scans (TR/TE 6.8/3.1 ms, flip angle 9°, matrix 244 × 227, voxel size 1.05 × 1.05 × 1.20 mm^3^).

Independent neuroradiologists at the UMCU evaluated all MRI scans; no gross morphological abnormalities were reported for any of the participants. All scans were coded and de-faced using the mri_deface function (Bischoff-Grethe et al., 2007) embedded in FreeSurfer (Fischl, 2012) to ensure the raters were always blind to subject identity during the analysis. Scans were inspected for overall quality of the image, no severe motion, scanner, or processing artefacts were detected.

### 2.3 Mammillary body volumes

**Fig. 1:**
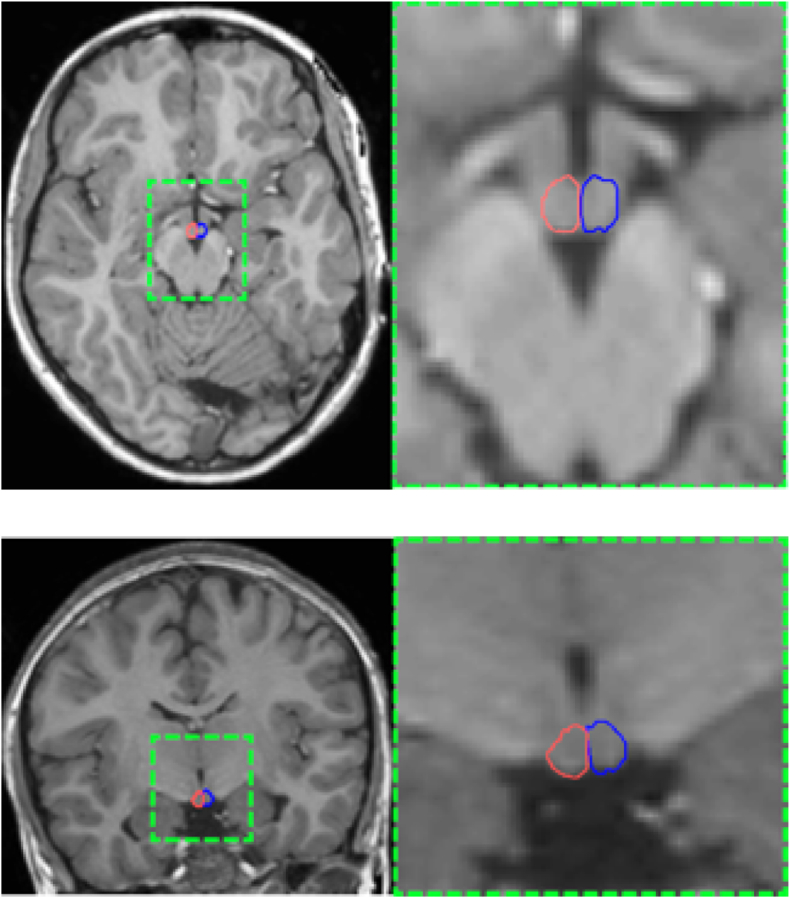
A typical example of contouring generated by the employed atlas-based solution. The figure shows the left (in red) and the right (in blue) mammillary bodies in an (a) axial and (b) coronal plane. The contours are shown in the context of the whole brain (left column) and at a higher magnification (x4; right column) for better visualization. (For interpretation of the references to colour in this figure legend, the reader is referred to the Web version of this article.)

Left and right MB volumes were evaluated using an in-house developed multi-atlas automatic segmentation solution. For this, all scans were re-sampled on a 0.5 × 0.5 × 0.5 mm grid prior to any volumetric analyses, to improve resolution and accuracy. To develop the multi-atlas segmentation solution, five representative scans were selected from each of the two databases and the MBs were outlined by an experienced pediatric radiologist. This resulted in two multi-atlas sets, one for each database, which were subsequently used to automatically segment the MBs for the remainder of the scans within each of the respective databases. The segmentation procedure was conducted in three stages. First, each of the scans in the database was linearly registered to the five scans within their assigned multi-atlas, estimating any potential translations, rotations and scaling in-between the image to be processed and the atlases. For this purpose, we employed the quadrature filter phase-based approach proposed by Hemmendorff et al. (2002) due to its previously demonstrated robustness to noise and inter-image intensity variations. This was then followed by a deformable image registration (DIR) stage, estimating morphological differences between the atlases and the scans being processed. This was performed via a variational DIR method called Evolution, initially proposed by Denis de Senneville et al. (Denis de Senneville et al., 2016). Since its introduction, the method has been successfully employed in the context of image-guided radiotherapy for multiple anatomical areas including brain, head-and-neck, lungs, upper and lower abdomen (Denis de Senneville et al., 2016; Zachiu et al., 2017, 2018, 2020) demonstrating an accuracy which is in-line with clinical recommendations. Thus, the displacements and deformations resulting from the linear and deformable image registration stages were used to propagate the five atlases onto the grid of the image being processed. In the final stage, a majority vote among the five propagated atlases was used to determine whether a particular voxel was classified as part of the left or right MB. Once the MBs were delineated, volumetric measures of the left and right MBs were obtained. An example of the delineations provided by the employed auto-contouring solution is shown in Fig. 1.

### 2.4 Statistics

The two datasets (neonates and children/young adults) were analyzed separately. First, left and right MB volumes were compared. Then, multivariable analysis was performed of total MB volume versus age and sex. In addition, in the neonatal dataset, the effect of prematurity on MB volume was assessed using a multivariable model with gestational age at scan, sex, and prematurity (yes/no) as independent variables. The effect of intracranial volume was assessed in the NICHE dataset. Multivariable analyses were performed using R-software. A p-value of 0.05 was considered significant.

## 3 Results

### 3.1 Laterality effects

#### 3.1.1 dHCP database

**Fig. 2:**
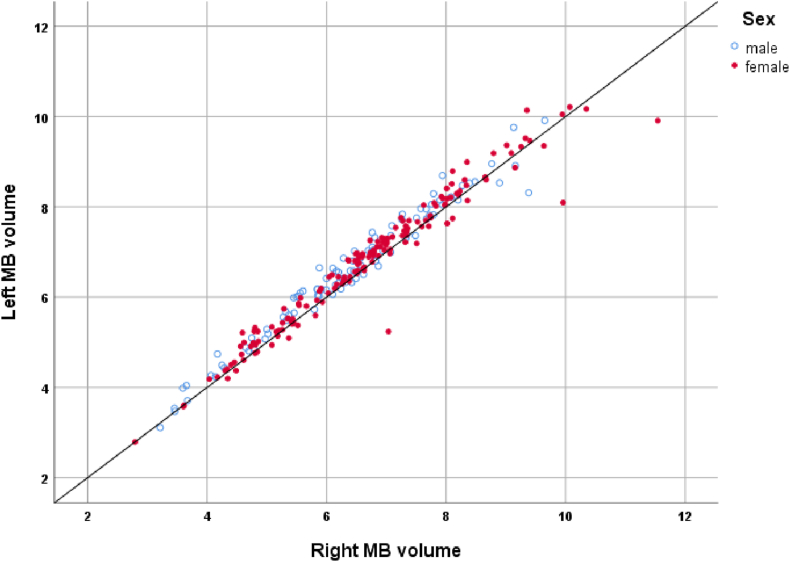
Relationship between left and right mammillary body volume in neonates (dHCP database). The line represents equal volumes.

In neonates, the left MBs were found to be significantly larger than the right MBs (p < 0.001*;* Fig. 2). This laterality effect was not dependent on age at scan or on sex (both p > 0.05). While there was an overall effect of hemisphere, the volume differences across hemispheres were very small; there was no incidence of obvious MB asymmetry at an individual level (see Fig. 2).

#### 3.1.2 NICHE database

**Fig. 3:**
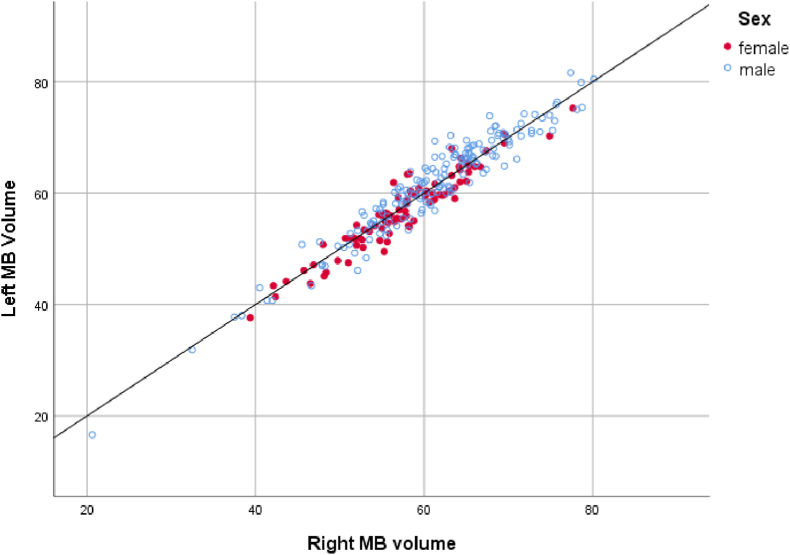
Relationship between left and right mammillary body volume in the 6-24-year-old group for males and females (NICHE database). The line represents equal volumes.

In older children and adults, the laterality effects were less clear (see Fig. 3). There was no overall effect of sex (p > 0.05) but the age at scan (and therefore MB volume), differentially affected the findings with the left MB being larger in when the MB volumes are >50 mm ^3^ (i.e., in older children). This may also reflect the distribution of participant age in the dataset. Again, there was no evidence of obvious MB asymmetry at an individual level. For this age range, MB laterality can be modelled as:

Left MB = −0.31542 + Right_MB * 1.00702.

### 3.2 Effects of age

#### 3.2.1 dHCP dataset

**Fig. 4:**
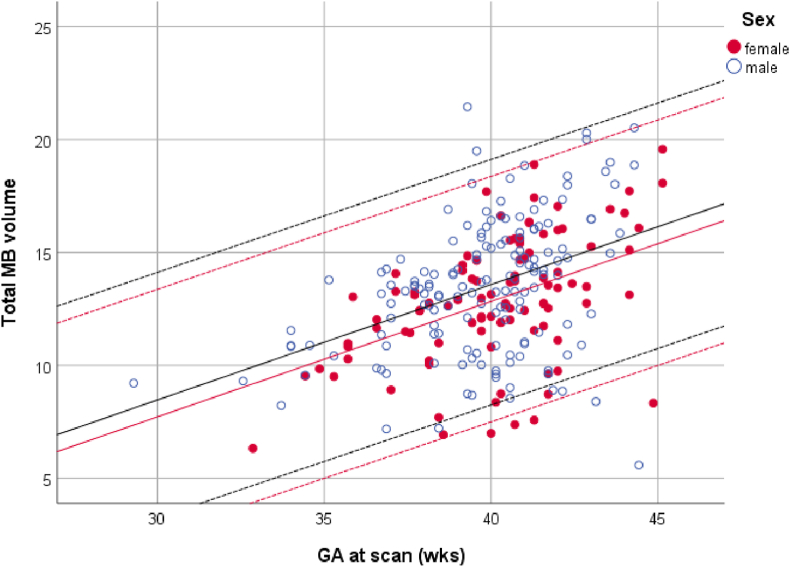
Relationship between gestational age at scan and total mammillary body volume (mm^3^). Lines of best fit and 95% confidence intervals are provided separately for males and females (dHCP database).

For neonates (gestational age at scan 29–46 weeks) the volume of both MBs combined increased by 0.51 mm^3^ per week (Fig. 4); this was reflected by a main effect of age (F_2,253_ = 28.95, p = 0.03). On average, the volume of boys was 0.75 mm^3^ larger than that of girls (p < 0.001). There was no effect of gestational age at birth or prematurity on MB volume at time of scan (p > 0.05).

#### 3.2.2 NICHE dataset

**Fig. 5:**
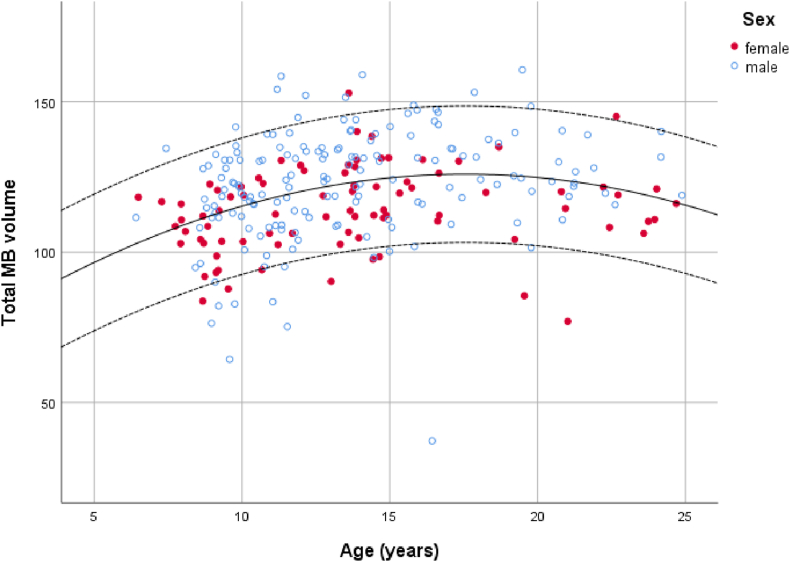
Relationship between age at scan and total mammillary body volume (mm^3^) for females and males. Lines represent the mean and 95% confidence intervals (NICHE database).

For the 6–24 age dataset there was an overall effect of age (t_2,248_ = 4.07, p < 0.001), with the peak occurring around 15 years, but no there was no effect of sex and no interaction (both p > 0.05; Fig. 5).

The growth trajectory of the MBs can be modelled using the following equation (age is in years):

Total MB volume in mm^3^ = 68.61 + 6.53 * Age −0.19 Age^2^

#### 3.2.3 Corrected mammillary body volumes – NICHE dataset

**Fig. 6:**
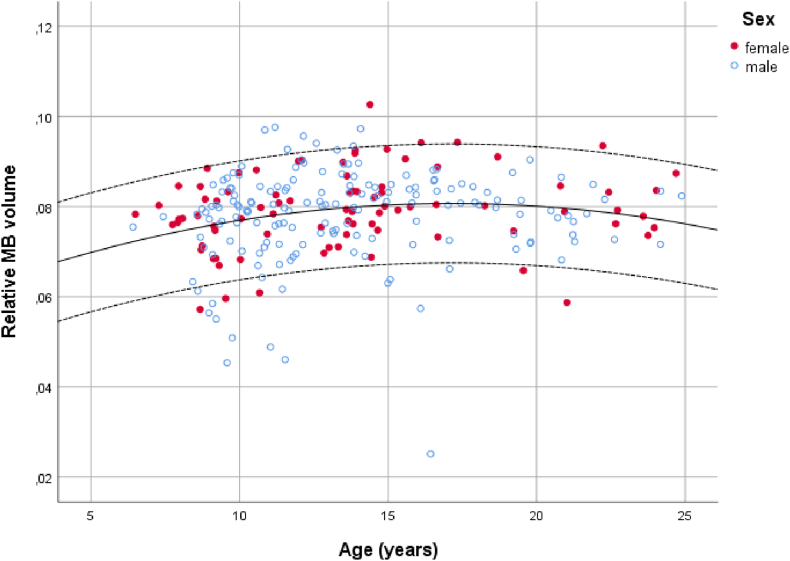
Relationship between age at scan and relative mammillary body volume (mammillary body volume corrected for intracranial volume) for males and females. Lines represent the mean and 95% confidence intervals (NICHE database).

When the MBs were corrected according to intracranial volume there was again an overall effect of age (t_2,248_ = 2.72, p < 0.01). The MBs increase in size, relative to overall intracranial volume, up to the age of approximately 12 years (Fig. 6). No sex effects were found using the corrected data set (p > 0.05).

The growth trajectory of the corrected MB volumes can be modelled using the equation (Age is in years):

Corrected MB volume = 0.059 + 2.52*10^−3^Age – 7.349*10^−5^ Age^2^

## 4 Discussion

In recent years, MB pathology has been increasingly found across wide-ranging neurological conditions (Meys et al., 2022). While it is well-known that the MBs are sensitive to thiamine deficiency, their sensitivity to hypoxia-ischemia has become more apparent, as shown by the prevalence of MB pathology in hypoxic-ischemic encephalopathy (Molavi et al., 2019; Lequin et al., 2021). In addition to acquired injury, MB pathology has also been associated with psychiatric and neurodevelopmental disorders (Küçükerden et al., 2022; Briess et al., 1998; Bernstein et al., 2012). To better understand the contribution of MB neuropathology in these diverse conditions, it is necessary to have a detailed structural knowledge of the MBs in normal populations. While normative data for the MBs has been reported for adults (Raz et al., 1992), and older adults in particular (Jin et al., 2020), the equivalent data for younger populations has not previously been available.

To address this gap in knowledge, we have produced a normative dataset of MB volumes for neonates and for children/young adult (6–24 years). These data were acquired from two databases: dHCP and NICHE. An automatic segmentation approach was developed in-house to obtain MB volumes from 506 T1-weighted scans acquired on 1.5T and 3T MRI scanners.

The MBs are relatively well-developed at birth (Koutcherov et al., 2003) but their overall size continues to increase with age, as shown in our current dataset. The rate of increase is most noticeable in the neonatal dataset which is consistent with previous studies showing the greatest rate of growth in the brain immediately after birth, with a 64% increase in overall brain volume during the first 3 months (Holland et al., 2014). For the later period, the MB volume continues to increase with age up until approximately 15 years. This is comparable to the growth pattern seen in the hippocampus whereas in the thalamus there is a slightly earlier peak volume (Wierenga et al., 2014a).

Both post-mortem and MRI studies have previously reported larger MBs in males than females (Bernstein et al., 2012; Jin et al., 2020; Sheedy et al., 1999); however, these studies did not correct for overall brain volume. In contrast, Raz et al. did control for skull size and found no sex-related differences (Raz et al., 1992). In our study we found the MBs to be smaller in females than males in the neonates, although this may be driven by differences in head-size. However, a previous study looking at brain size in neonates found no overall differences in sex at birth, although there was a greater growth trajectory in males versus females during the first three months of life (Holland et al., 2014). In contrast, no sex differences were found for the children/young adults when either comparing raw volumes or when the MB volumes were corrected for intracranial volume. This lack of differences across males and females contrasts with the findings from other subcortical structures from the NICHE dataset (e.g., hippocampus and thalamus) where clear sex differences were found (Wierenga et al., 2014a).

There was an indication of lateralization effects in the MBs, with the left MB being larger than the right. This effect was most noticeable during the neonatal period but was also evident in the 6–24 age group when MB volumes were greater than 50 mm^3^. This may reflect the data distribution in the older dataset rather than a genuine change over age. Very few previous studies have assessed lateralization in the MBs. Jin et al. found no hemispheric differences in the MBs of older individuals with or without mild cognitive impairment (Jin et al., 2020). However, there were only 47 individuals in each subgroup so it is possible that, given the hemispheric differences we observed were quite small, larger datasets are required for hemispheric differences to emerge. It is also not known whether the lateralization effects that we have reported are driven by handedness. At present, there are variable findings concerning lateralization effects in subcortical regions. A large-scale meta-analysis of studies predominantly involving adults, found the thalamus to be larger in the left hemisphere and the hippocampus larger in the right (Guadalupe et al., 2017). In contrast, in the first three months of life, both the hippocampus and thalamus were found to be larger on the right (Holland et al., 2014). Further studies are needed to determine the extent of lateralization in subcortical regions, how this develops over age and how this may contribute to function.

MB asymmetry has been reported as occurring in 6–14% of the normal population (Ozturk et al., 2008; Tagliamonte et al., 2015). However, these studies used a qualitative approach to classify the MBs as asymmetric, depending on their appearance. While we have found population-level lateralization effects, with the left MB slightly larger than the right, these volume differences are not large enough to be noticed when visually examining the scans. This suggests that previous reports of asymmetry do not necessarily reflect differences in MB volume but instead reflect MB displacement or are due to the position of the head in the scanner.

One limitation of the study is that we only had our own data for the ages of 6–24 years and therefore used a publicly available dataset to acquire the volumetric data for neonates. This could have affected selection criteria and methodological differences across the age groups. However, given the large number of cases that have been included, the data presented should be robust. A second limitation is that due to the use of separate, non-overlapping datasets, there is a gap in data from the neonatal period to age 6. Given the likely differential rates of growth during this time period, it is difficult to extrapolate the missing data from the data we have available.

A further limitation is that the MBs are very small, particularly in younger populations, making them hard to delineate and accurately measure automatically; but automatic measurements are necessary to produce large reliable datasets. To address this, we developed a volumetric tool that automated parts of the process while still requiring manual input for optimization. This has enabled data to be obtained for a large number of individuals while still ensuring the measures are as accurate as possible for such a small structure.

We have provided normative datasets of MB volumes for neonates and 6–24-year-olds. MB volume increases with age and this is particularly noticeable during the neonatal period. The left MBs are slightly larger than the right, again this is most apparent in neonates but the same pattern is found in older children/young adults. While MBs in males were larger during the neonatal period there were no sex differences in the older age group. This dataset could help identify individuals whose MB volumes fall outside of the normal limits so that they can be provided with additional support and follow-up **care** if needed. Having a better understanding of expected size and growth trajectory in normal populations can help us understand how any preventions or modifiers affect MB volume loss; this is particularly relevant for studies looking at pre- and post-operative effects as well as long-term effects of radiotherapy, both of which can impact the MBs.

## Data sharing

The data were acquired from two databases. The first is the developing Human Connectome Project which is publicly available. The second is the NICHE database. The measurements acquired can all be obtained on request. The scans themselves cannot be made available due to ethical/consent limitations.
